# Solar-light photocatalytic disinfection using crystalline/amorphous low energy bandgap reduced TiO_2_

**DOI:** 10.1038/srep25212

**Published:** 2016-04-28

**Authors:** Youngmin Kim, Hee Min Hwang, Luyang Wang, Ikjoon Kim, Yeoheung Yoon, Hyoyoung Lee

**Affiliations:** 1 Center for Integrated Nanostructure Physics, Institute for Basic Science, Department of Chemistry and Department of Energy Science, Sungkyunkwan University, Suwon 440-746, Korea

## Abstract

A generation of reactive oxygen species (ROS) from TiO_2_ under solar light has been long sought since the ROS can disinfect organic pollutants. We found that newly developed crystalline/amorphous reduced TiO_2_ (rTiO_2_) that has low energy bandgap can effectively generate ROS under solar light and successfully remove a bloom of algae. The preparation of rTiO_2_ is a one-pot and mass productive solution-process reduction using lithium-ethylene diamine (Li-EDA) at room temperature. Interestingly only the rutile phase of TiO_2_ crystal was reduced, while the anatase phase even in case of both anatase/rutile phased TiO_2_ was not reduced. Only reduced TiO_2_ materials can generate ROS under solar light, which was confirmed by electron spin resonance. Among the three different types of Li-EDA treated TiO_2_ (anatase, rutile and both phased TiO_2_), the both phased rTiO_2_ showed the best performance to produce ROS. The generated ROS effectively removed the common green algae *Chlamydomonas*. This is the first report on algae degradation under solar light, proving the feasibility of commercially available products for disinfection.

Around the globe, various parts of the regional aquatic ecosystems, including marine and fresh water, are being damaged by an increase in the frequency and severity of algal blooms, which are caused by natural phenomena or nutrient pollution from human activities such as agriculture, wastewater, and the use of fossil fuels. As we know, among the thousands of different algae, only a few species are known to be harmful and toxic. Harmful algal blooms like *Microcystis* and *Anabaena* can produce microcystins (cyanoginosins), which are dangerous toxins that can cause serious damage to the liver and/or sicken or even kill humans and animals^1^,^2^. Even though most algae are nontoxic, these species can also cause huge economic damage from fish kills, due to a competitively limited amount of oxygen in the water, as well as cause environmental problems if the algae are overgrown and massively accumulated in an aqueous medium. Most algae blooms lead to severely negative effects on the environment, economy, and public health^3^. Thus, to eliminate or reduce the algae blooms, there have been numerous approaches such as dilution and flushing, hypolimnetic withdrawal, activated sludge, phosphorus inactivation, oxidants (Cl_2_, ClO_2_), sediment oxidation, biomanipulation, and algicide (biocide) application (CuSO_4_)^1^,^4^. However, these methods have not been significantly effective to reduce the algae blooms, and some methods cause secondary pollution to aquatic ecosystems by causing other problems or are culturally undesirable.

In recent decades, advanced photocatalyst semiconductors using physico-chemical processes have been applied with promising results for purification methods such as the removal of organic contaminants, deodorization, the disinfection of surfaces, and the removal of polluted air and water^2^,^3^,^5^,^6^,^7^,^8^,^9^. Studies on semiconductor materials that are abundant, stable, and efficient for photocatalytic reactions are still attracting interest worldwide because photocatalytic technology using solar energy has been considered to be a superior approach to solve both energy and environmental problems compared to other methods. Among many other semiconductor nanomaterials, titanium dioxide (TiO_2_) is well known for its photocatalytic properties and is considered to be the most suitable candidate, mainly due to its cheapness, worldwide availability, chemical stability, high photocorrosion stability, and environmental friendliness^9^,^10^. TiO_2_ also has good properties in charge transport, photo-electronic generation, light scattering, and energy conversion. When TiO_2_ is irradiated by light that has a wavelength equal to or smaller than its energy bandgap (3.2 eV or 387 nm), electron/hole pairs can be generated and can migrate to the crystalline surfaces. In the presence of oxygen, these electron/hole pairs can be reacted in an aqueous medium to generate reactive oxygen species (ROS), including hydroxyl radicals (OH·) and superoxide anions (O_2_^−^·), which are capable of degrading organic pollutants including algae and bacteria^11^,^12^,^13^,^14^. Despite its many advantages, conventional TiO_2_ under a natural resource, such as sunlight, has intrinsically limited abilities for generating hydroxyl radicals (OH·) and superoxide anions (O_2_^−^·), mainly due to its large energy bandgap (3.2 eV or more), which leads to poor optical performance in the visible and near-infrared regions. This large energy bandgap characteristic of the conventional TiO_2_ is the major drawback for its practical applications using sunlight. Thus, many researchers have struggled to overcome the large bandgap limitation of the conventional TiO_2_ by applying different strategies such as doping with certain elements (cationic or anionic impurities)^11^,^15^,^16^,^17^, using TiO_2_ nanocomposites, and the bottom-up synthesis^18^ of TiO_2_ to control the nanocrystal structure or to modify its electronic band structure. Recently, hydrogenated TiO_2_ (H-TiO_2_) or black TiO_2_ that is capable of absorbing visible and infrared light has gained much attention in the field of photocatalytic applications^19^,^20^,^21^. This H-TiO_2_ shows superior performance in comparison with previous TiO_2_ materials in terms of photocatalytic activity. The reduced energy bandgap property of the H-TiO_2_ could be explained due to the formation of Ti-H and Ti-OH bonds and the high concentration of defects^22^. Despite its merits, the absorption enhancement has not been very effective regarding the visible-light photocatalytic activity. One of the greatest disadvantages for the use of the H-TiO_2_ materials is the troublesome preparation method, which requires extreme reaction conditions using a high pressure (20 bars) hydrogen gas and a high reaction temperature.

Therefore, it is highly necessary to find a new way to prepare the low energy bandgap TiO_2_ materials, including reduced TiO_2_, under milder reaction conditions such as room temperature, and also to provide a method for mass production. It is expected that a low energy bandgap TiO_2_ like a reduced TiO_2_ (rTiO_2_) material can effectively generate electrons/holes under sunlight, enhance the production of the ROS (1, 2), and as a result, effectively disinfect algae blooms. Unfortunately, to date there have been no reports on the killing or effective reduction of algae using the low energy bandgap TiO_2_ under sunlight.


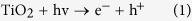



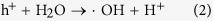


Herein, we demonstrate the novel disinfection of freshwater algae using the crystalline/amorphous low energy bandgap rTiO_2_ materials including a reduced rutile-only phased and both a crystalline anatase/amorphous reduced rutile-phased rTiO_2_ (called HYL’s rTiO_2_) under a solar light system. First, we briefly introduce how to synthesize the HYL’s rTiO_2_ materials that are reduced with a solution-processible lithium-ethylenediamine (Li-EDA) solution at room temperature. Then, we report on the photocatalytic ability of the several HYL’s rTiO_2_ materials via the ROS generation with UV or solar or visible light irradiation and finally, on the disinfection of freshwater algae with sunlight, which can guide the practical environmental application for the reduction of algae blooms in freshwater rivers with our solar light system.

## Results

### Crystal structure characterization of TiO_2_ samples

**Figure 1:**
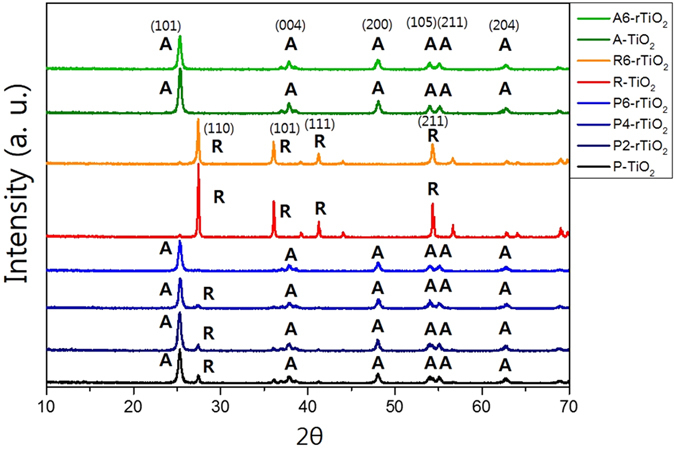
X-ray diffraction data of TiO_2_ samples (P-TiO_2_: pristine P25 TiO_2_; P2-rTiO_2_: TiO_2_ after two days of being reduced; P4-rTiO_2_: TiO_2_ after four days of being reduced; P6-rTiO_2_: TiO_2_ after six days of being reduced; R-TiO_2_: rutile phase only TiO_2_; R6-rTiO_2_: rutile phase only TiO_2_ after six days of being reduced; A-TiO_2_: anatase phase only TiO_2_; A6-rTiO_2_: anatase phase only TiO_2_ after six days of being reduced).

Figure 1 shows the XRD patterns of TiO_2_ materials that were reduced over different reaction times (from two to six days). Rutile phase only TiO_2_ (R-TiO_2_) and anatase phase only TiO_2_ (A-TiO_2_), and their corresponding reduced samples after being reduced for six days (R6-rTiO_2_ and A6-rTiO_2_, respectively) are shown as well. The main peaks at 25.3°, 37.8°, 48.1°, 53.9°, 55.1°, and 62.7° correspond to the XRD pattern of the anatase phase, and the main peaks at 27.4°, 36.1°, 41.3°, and 54.3° correspond to the XRD pattern of the rutile phase^7^,^16^,^23^,^24^. The pristine P25 TiO_2_ (P-TiO_2_) and its corresponding reduced samples after being reduced for two days (P2-rTiO_2_), four days (P4-rTiO_2_), and six days (P6-rTiO_2_) are shown. P-TiO_2_ is known to contain both phases (anatase:rutile = 70~80%:30~20%). Surprisingly, when the P-TiO_2_ was reduced by the Li-EDA to give HYL’s rTiO_2_, the peak intensities of the HYL’s rTiO_2_ at 27.4°, 36.1°, and 41.3° were decreased, which means that the rutile phase was reduced as the reaction time was increased. We like to refer that the possible mechanism on why the rutile phase is selectively (or easily) reduced in comparison to the anatase phase can be due to the large gap in the protonation constants between rutile phase and anatase phase against pH conditions^25^. We calculated the percentage of antase and rutile with various reaction times through the highest peak of each anatase phase peak (25.3°) and rutile phase peak (27.4°). The rutile % of P-TiO_2_, P2-rTiO_2_, P4-rTiO_2_ and P6-rTiO_2_ in comparison with anatase had 22.5, 16.6, 11.9 and 3.93%, respectively.

**Figure 2:**
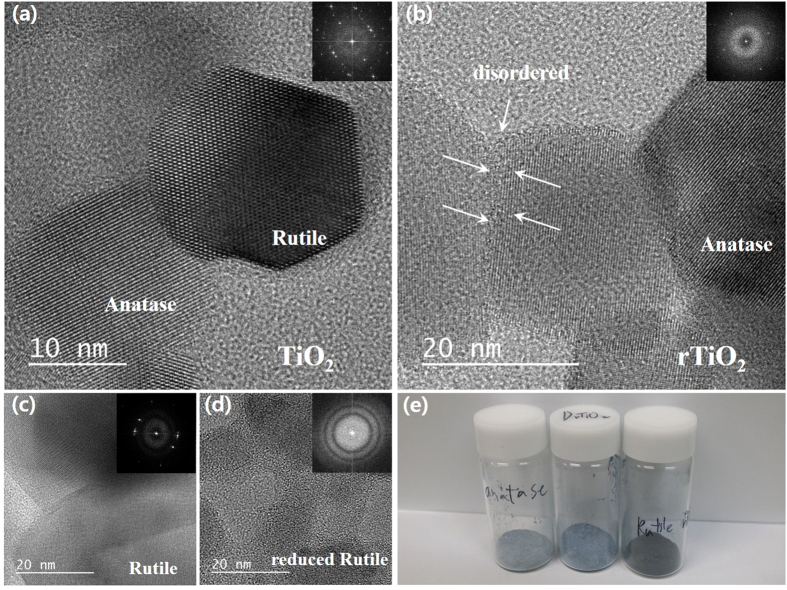
High resolution TEM images of TiO_2_ samples. (**a**) Pristine P25 TiO_2_ (P-TiO_2_) showing an anatase-rutile interface, (**b**) TiO_2_ after six days of being reduced (P6-rTiO_2_) showing a disordered structure and ordered anatase phase, (**c**) rutile phase only TiO_2_ (R-TiO_2_) and (**d**) rutile phase only TiO_2_ after six days of being reduced (R6-rTiO_2_), with nanoparticles showing a disordered structure. (**e**) The photo images of anatase phase only TiO_2_ after six days of being reduced (A6-rTiO_2_, left), P6-rTiO_2_ (middle), and R6-rTiO_2_ (right).

Figure 2(a–d) shows the high resolution TEM images of the TiO_2_ samples, and the insets are the associated SAED patterns corresponding to each sample. The actual crystallite size of the P-TiO_2_ was about 15 to 50 nm. We observed that the crystallinity of the P6-rTiO_2_ and R6-rTiO_2_ had been altered. The Li-EDA-treated reduction of the R-TiO_2_ and P-TiO_2_ caused the rutile phase region to become disordered so that the color of the R-TiO_2_ turned black, like that of the H-TiO_2_, while that of the anatase phase was changed only slightly. The TEM image of P-TiO_2_ shows the well-defined anatase–rutile interface (Fig. 2a). However, the TEM image of the P6-rTiO_2_ shows the contact boundary between the ordered and disordered TiO_2_ nanoparticles (Fig. 2b). In Fig. 2c,d, we could observe dramatic changes in the rutile crystal structure after reduction. As a result, the colors of the A6-rTiO_2_, R6-rTiO_2_, and P6-rTiO_2_ were all different. After reduction, a light blue color for the A6-rTiO_2_, dark black color for the R6-rTiO_2_, and deep blue color for the P6-rTiO_2_ samples are shown in Fig. 2e, while all of them were white before being treated with Li-EDA.

**Figure 3:**
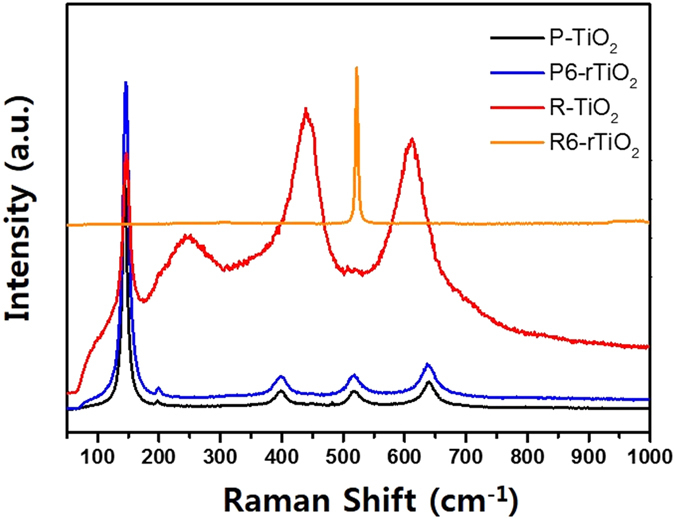
Raman spectra of pristine P25 TiO_2_ (P-TiO_2_) and rutile phase only TiO_2_ (R-TiO_2_) and their reduced samples (P6-rTiO_2_ and R6-rTiO_2_).

Figure 3 shows the Raman spectra of P-TiO_2_ and R-TiO_2_. Those of P6-rTiO_2_ and R6-rTiO_2_ are also displayed. P-TiO_2_ and P6-rTiO_2_ showed almost the same peaks at 149, 400, 520, and 637 cm^−1^, which are assigned to the *E*_*g*_, *B*_*1g*_, *A*_*1g*_ *& B*_*2g*_, and *E*_*2g*_ modes of the anatase phase, respectively^25^. The Raman peaks of R-TiO_2_ that are shown at 448 and 612 cm^−1^ were assigned to *B*_*1g*_ and *B*_*2g*_, respectively. However, the Raman peaks of R-TiO_2_ completely disappeared for the R6-rTiO_2_ that was reduced by treatment with Li-EDA, which means that the molecular structure of the rutile phase was dramatically changed by the Li-EDA reducing agent.

### Chemical properties of TiO_2_ samples

**Figure 4:**
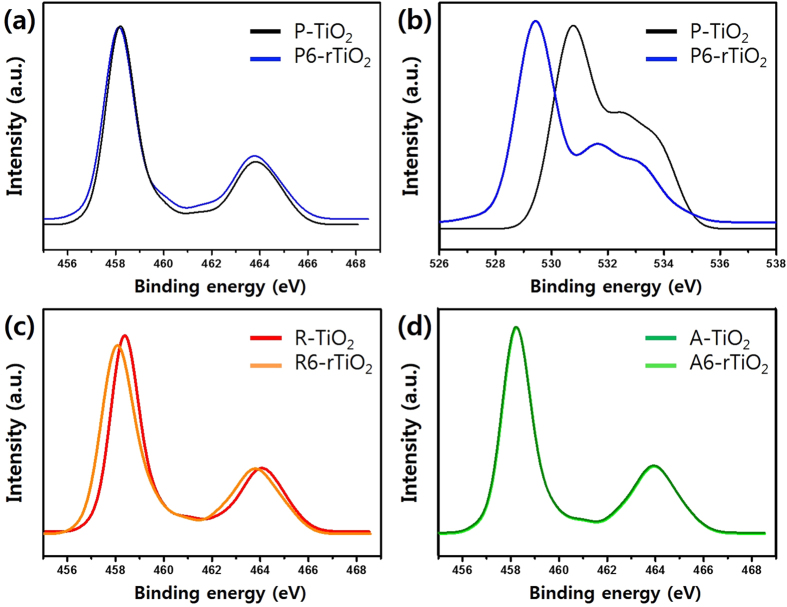
X-ray photoelectron spectra of TiO_2_ samples. (**a,b**) Ti 2p and O 1s XPS spectra of the pristine P25 TiO_2_ (P-TiO_2_) and its reduced sample (P6-rTiO_2_); (**c,d**) Ti 2p XPS spectra of the rutile phase only TiO_2_ (R-TiO_2_), anatase phase only TiO_2_ (A-TiO_2_), and their reduced samples (R6-rTiO_2_ and A6-rTiO_2_, respectively).

Figure 4 shows the XPS spectra of the P-TiO_2_, R-TiO_2_, and A-TiO_2_, as well as their reduced samples, P6-rTiO_2_, R6-rTiO_2_, and A6-rTiO_2_. Each oxidation state was analyzed based on the Ti 2p and O 1s core levels to investigate the change in the chemical bonds of the P6-rTiO_2_ compared to the P-TiO_2_ sample. The original Ti 2p exhibited two peaks of 458.2 eV and 463.8 eV, which correspond to 2p3/2 and 2p1/2, respectively^26^,^27^. The P6-rTiO_2_ revealed a similar behavior, but was shifted slightly toward the lower binding energy of Ti 2p peaks at 458.1 eV (2p3/2) and 463.7 eV (2p1/2). Both peaks are assigned to the Ti^4+^ oxidation state in TiO_2_ crystal, which is typical according to the previously reported XPS data^17^,^26^,^28^,^29^. However, the single O 1s peak, which can be assigned to the Ti-O bond at 530.7 eV of the P-TiO_2_, was shifted to 529.4 eV (−1.3 eV) for P6-rTiO_2_. This negative shift indicates that a different bonding environment such as oxygen vacancies occurred, making an electron negativity difference to the original value possible by the change in a surface chemical bond induced by the Li-EDA reduction. Surprisingly, the XPS peaks of the Ti 2p for A-TiO_2_ and A6-rTiO_2_ were almost identical, but the XPS peaks of the Ti 2p for the R-TiO_2_ at 458.4 eV (2p3/2) and 464.1 eV (2p1/2) were negatively shifted to 458.1 eV (2p3/2) and 463.8 eV (2p1/2), respectively, which indicates that the reduction of TiO_2_ with Li-EDA occurred only in the rutile phase, but not in the anatase phase of TiO_2_. We also performed XPS survey scan spectra (see Supporting Information Figure S1) and EDS analysis of reduced TiO_2_ samples (see Supporting Information Figure S2) to exclude the possibility of contamination such as Li or Cl in our samples.

**Figure 5:**
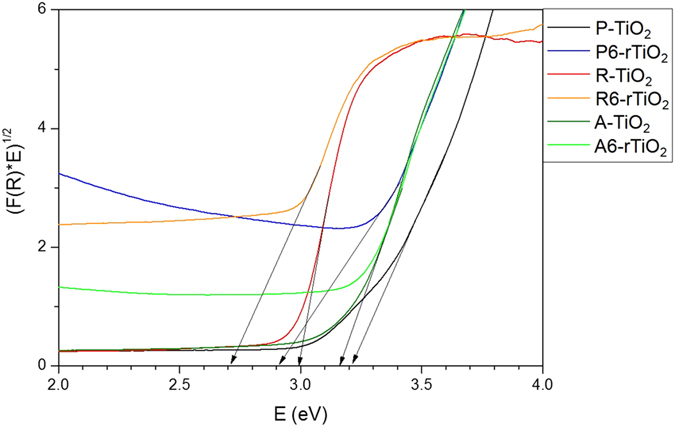
Transformed Kubelka−Munk function versus the photon energy graphs of the TiO_2_ materials and their reduced TiO_2_ samples.

To obtain a bandgap (Eg), the optical absorption of the TiO_2_ nanomaterials is obtained from the UV-vis absorption spectra via a diffuse reflectance spectra using the Kubelka-Munk function,


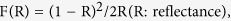


with the calculation of the photon energy given by E = hν = 1240/λ; the transformed Kubelka-Munk spectra are shown in Fig. 5. The dry TiO_2_ powder sample was used to avoid difficulties, even though TiO_2_ can be dispersed in solvent. The intersection between the linear fit and the photon energy (E, eV) axis gives the value of the bandgap (Eg)^17^,^30^,^31^. In this way, the bandgaps of the TiO_2_ samples are indicated in Fig. 5. A reduction of the P-TiO_2_ (Eg = 3.2 eV) gives the P6-rTiO_2_ (Eg = 2.9 eV), and the R-TiO_2_ (Eg = 3.0 eV) yields the R6-rTiO_2_ (Eg = 2.7 eV). However, the bandgap reduction of the A-TiO_2_ did not occur, and as a result, the bandgap was not changed (for A-TiO_2_ and A6-rTiO_2_, Eg = 3.16 eV). We also took Valence band XPS spectra of TiO_2_ samples (see Supporting Information Figure S3) to estimate the valence band maxima. Thin black lines of each spectra show the linear extrapolation of the curves used for deriving the band edge position of TiO_2_ samples. The valence band maxima of P-TiO_2_, P3-rTiO_2_, P6-rTiO_2_, R-TiO_2_ and R6-rTiO_2_ are of 1.6, 1.8, 2.1, 1.9 and 2.2 eV below the Fermi energy, respectively. Considering the bandgap changes of each sample with the diffuse reflectance spectra, the proposed band diagram of TiO_2_ samples are shown in Figure S4.

**Figure 6:**
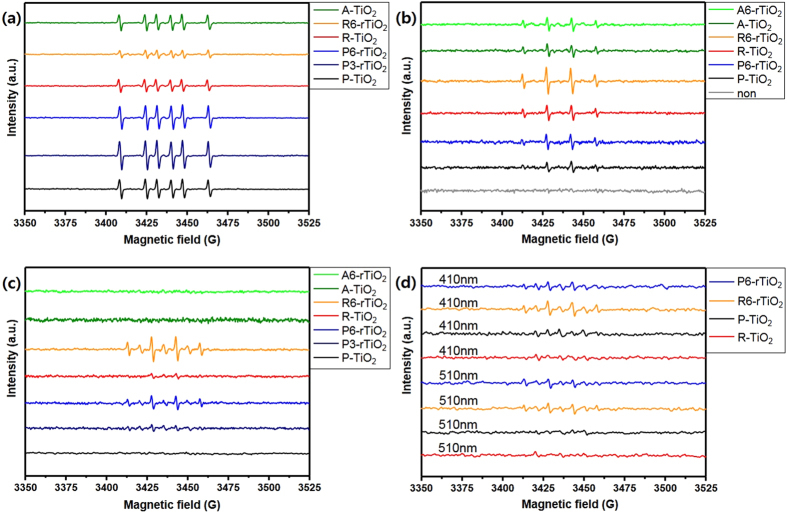
Electron spin resonance data of each TiO_2_ sample under different conditions (A-TiO_2_: anatase phase only TiO_2_; A6-rTiO_2_: anatase phase only TiO_2_ after six days of being reduced; R-TiO_2_: rutile phase only TiO_2_; R6-rTiO_2_: rutile phase only TiO_2_ after six days of being reduced; P-TiO_2_: pristine P25 TiO_2_; P3-rTiO_2_: TiO_2_ after three days of being reduced; P6-rTiO_2_: TiO_2_ after six days of being reduced) (**a**) in ethanol and water co-solvent (H_2_O:EtOH = 5:1) under UV irradiation, (**b**) in water under UV irradiation, (**c**) in water under solar irradiation, and (**d**) in water under irradiations at 410 and 510 nm wavelength using XBPA410, XBPA510 bandpass filter, UniNanoTech Co., Ltd.

To investigate the photo-excitation and generation of the reactive oxygen radicals from the TiO_2_ nanomaterials, ESR (also called electron paramagnetic resonance) was measured as shown in Fig. 6. The ESR spin-trap technique in water, accomplished using 5,5-dimethyl-1-pyrroline *N*-oxide (DMPO) as a spin trap^32^ at room temperature, can detect the hydroxyl radical that is produced from the oxidation of water by the photogenerated holes from the TiO_2_ nanomaterials^33^. All TiO_2_ samples were prepared as follows. First, 5 mg of each TiO_2_ powder were dispersed and sonicated in 4 ml of DI water in a small vial, and we then took 1 ml of the dispersion mixture, added approximately 4 μl of DMPO, placed the resulting combination into another small vessel, and vigorously shook it. The samples were irradiated for 5 min by a UV lamp or solar simulator. The spectra in Fig. 6a, in comparison with those in Fig. 6b,c, are of samples in a different solvent system in which ethanol was added (5 mg of each powder in 4 ml of mixed solvents, H_2_O:EtOH = 5:1) to confirm the production of free hydroxyl radicals. The addition of ethanol quickly converted the hydroxyl radicals into carbon (C)-centered radicals. These spectra can be assigned to DMPO spin adducts of C-centered radicals^32^. The spectra in Fig. 6b of the samples in water under UV irradiation are composed of signals in a set with four characteristic peaks with the intensity ratio of 1:2:2:1, which are from hydroxyl radical spin adducts that are assigned to DMPO-•OH spin adducts^33^,^34^. ROS can be seen for all samples except the control, which is just DMPO in water (non). The ESR spectra for samples irradiated by the solar simulator in water are shown in Fig. 6c. Generally speaking, as the photons’ energy decreased in the irradiation from the UV to the solar light system, the ESR peaks decreased or completely disappeared. This phenomenon is due to the fact that ESR spectra could be observed when the TiO_2_ samples were irradiated with photons of energy exceeding the bandgap energy. In contrast, the intensity of the hydroxyl radicals increased as the surface oxygen vacancies increased, resulting in better photocatalytic activity. As a result, the R6-rTiO_2_ and the P6-rTiO_2_ (having both anatase and rutile phases) exhibited strong ESR peaks under solar light, but the other non-reduced TiO_2_ samples and A6-rTiO_2_ did not show any ESR signals as shown in Fig. 6c. We also checked ESR for R6-rTiO_2_ and the P6-rTiO_2_ under visible radiations at 410 and 510 nm using bandpass filter (XBPA410, XBPA510 bandpass filter, UniNanoTech Co., Ltd.) as shown in Fig. 6d. The reduced R6-rTiO_2_ and the P6-rTiO_2_ samples showed trace of radical peaks, but the radical peaks of the non-reduced samples were hardly observed. In a partial conclusion, with the active visible light of the solar light, the R6-rTiO_2_ and the P6-rTiO_2_ can generate the ROS, which is directly applicable for our global environmental problems.

### Algae disinfection property of rTiO_2_

**Figure 7:**
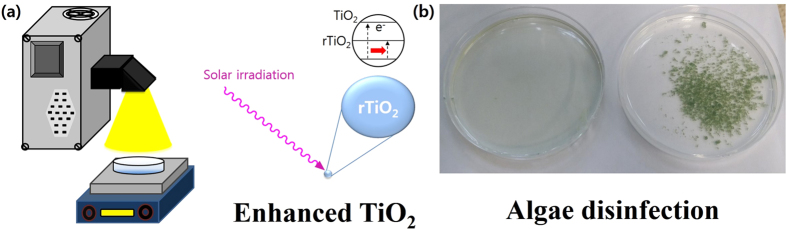
(**a**) The photocatalytic activity of reduced TiO_2_ and its experimental setup. (**b**) Two petri dishes containing algae samples before (left, well dispersed) and after (right, aggregated and precipitated) the disinfection with reduced TiO_2_.

**Figure 8:**
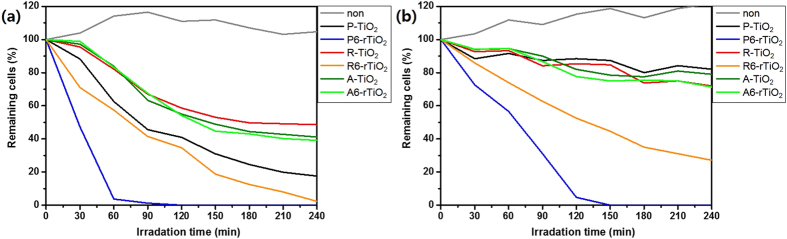
Photocatalytic degradation graph comparison of algae with each TiO_2_ sample after exposure to (**a**) UV light or (**b**) solar light.

Figure 7 illustrates the image of the experimental setup and the photocatalytic activity of the rTiO_2_, as detailed in the Experimental Section, and Table 1 shows the experimental conditions for the TiO_2_ samples. The photo-activity of all TiO_2_ samples was evaluated by observing the degradation of a *Chlamydomonas segnis* (*Lobochlamys segnis,* NLP Co., Ltd) suspension under UV light or solar light irradiation, as shown in Fig. 8. In Fig. 8a, after 4 h of UV irradiation, the percentages of cells remaining for the control (non, only algae), P-TiO_2_, R-TiO_2_, R6-rTiO_2_, A-TiO_2_, and A6-rTiO_2_ were 104.7, 17.5, 48.6, 2.3, 41.1, and 39.1%, respectively. The percentage of remaining cells is compared to the initial number of algal cells. The R6-rTiO_2_ degraded 50% of the cells after 1.5 h and had degraded almost all of them after 4 h. Amazingly, the P6-rTiO_2_ had almost fully degraded the algae cells after 1 h and had completely degraded them after 2 h, while the P-TiO_2_ degraded 60% of the algae. In Fig. 8b, after 4 h of solar irradiation, the percentages of cells remaining for the non, P-TiO_2_, R-TiO_2_, R6-rTiO_2_, A-TiO_2_ and A6-rTiO_2_ samples were 121.5, 82.1, 72.0, 27.1, 79.0, and 71.3%, respectively. The R6-rTiO_2_ had degraded about 50% after 2 h, whereas most of the other TiO_2_ samples showed a low activity. Surprisingly, P6-rTiO_2_ had almost fully degraded the algae after 2 h and had completely degraded them after 2.5 h. In fact, the unreduced samples, such as P-TiO_2_, R-TiO_2_, and A-TiO_2_, were active only under UV light, but were not effective under solar light. However, the Li-EDA-treated TiO_2_ (P6-rTiO_2_ and R6-rTiO_2_) showed effectiveness, even under solar light. In partial conclusion, the photocatalytic activities of the TiO_2_ samples sharply increased with Li-EDA reduction, but only the P6-rTiO_2_ nanomaterial could completely kill the algae under UV, and even under solar light. These breakthrough photocatalytic performances can be easily explained due to the reduced TiO_2_ nanomaterials that permit the creation of oxygen vacancies (defects) that work as trapped holes^3^,^18^,^35^, leading to the lower recombination of electrons and holes, even with the low energy bandgap irradiation. In addition, the R6-rTiO_2_, which has a lower energy bandgap (2.7 eV) than that of the P-TiO_2_ (3.2 eV), could generate electrons, even with the low-energy irradiation such as solar light, which is composed of most visible and infrared wavelengths as well as UV light. As a result, the newly generated electrons can produce ROS and effectively degrade the algae under sunlight. Without the low energy bandgap of rTiO_2_, the electrons could not be produced under the relatively weaker solar light. Furthermore, we found that the TiO_2_ samples containing both reduced rutile and anatase phases exhibited higher catalytic activity than that of the one-phase TiO_2_ samples, including the R6-rTiO_2_ or A6-rTiO_2_. This may be because the reduced rutile phase part of P6-rTiO_2_ that has an irregular and disordered phase easily generates electrons/holes under solar light, and simultaneously, the unreduced anatase crystalline phase part of P6-rTiO_2_ can enhance the charge separation of the electrons/holes by quickly transporting the electrons or the holes^19^,^35^,^36^. The possible mechanism about the charge transportation and separation efficiency was already reported in our group with the time-correlated single-photon counting (TCSPC) and PL experiments. TCSPC result revealed that the disordered/ordered TiO_2_ phases gave the highest exciton separation and enhanced photo-catalytic process, and also PL quenching schematic illustration showed Li-EDA treatment can critically block the electron loss by obstructing the recombination^25^. This phenomenon is more effective under solar light irradiation. As a result, both the reduced irregular rutile phase and the unreduced anatase crystalline phase perform best for the disinfection of algae blooms even under sunlight, which is the first time that TiO_2_ has been used for the disinfection of algae under sunlight, which can be applicable to our rivers under real-world conditions. These remarkable optoelectronic properties of rTiO_2_, which has both reduced rutile and unreduced anatase phases, could provide valuable insight for various applications such as solar cells, electronic devices, and photocatalysis.

## Discussions

The analysis of XRD, TEM and Raman clearly demonstrate the crystallinity change of rTiO_2_ and the analysis of XPS, DRS and ESR show large differences especially regard to chemical properties and as a photocatalyst.

In conclusion, we demonstrated the simple room temperature one-pot synthesis of reduced TiO_2_ from pristine TiO_2_. This synthetic method could also be used in mass production, and the resulting reduced TiO_2_ was composed of both disordered rutile and ordered anatase phases. The reduced TiO_2_ showed the highest photo-activity not only under UV irradiation, but also under solar irradiation in the generation of reactive oxygen radicals, such as hydroxyl radicals that are capable of disinfecting algae. Thus, the reduced TiO_2_ is superior to previously reported TiO_2_ nanoparticles in generating ROS at lower energy levels. We assume that this improved photocatalytic performance is due to the oxygen defects of the TiO_2_ surface that functioned as trapped holes, leading to a lower recombination of electrons and holes with a reduction of their bandgap. We also found the multi-phase TiO_2_ to have better catalytic activity than the mono-phase TiO_2_, especially under solar light irradiation. For this reason, we found that the multi-phase rTiO_2_ was more effective for the degradation of algae than the single phase crystal forms of TiO_2_, because the electrons generated from the reduced rutile part easily moved to the anatase part of TiO_2_, leading to the higher production of ROS and resulting in better disinfection of the algae. This phenomenon was more apparent under sunlight than it was under UV light. Furthermore, rTiO_2_ nanomaterials, having a small band gap in comparison with normal TiO_2_, can be applied in various optoelectronic devices, including photovoltaic cells and other energy applications.

## Methods

### Materials and preparation of reduced TiO_2_

Pristine TiO_2_ was purchased commercially from Degussa (P25); lithium (Li) and ethylenediamine (EDA) were purchased from Sigma Aldrich. The purchased reagents were used directly with no further purification. The rTiO_2_ nanoparticles were prepared as follows: 500 mg of TiO_2_ (10 mg/ml) powder were dispersed in 50 ml of ethylenediamine, and then Li (0.347 g) was added to make a concentration of 1 mol/L. The solution was sonicated for 15 min and stirred at room temperature for two to six days. All of the procedures were done in anhydrous and nitrogen atmosphere conditions. As the reaction proceeded, the solution changed in color from white to dark blue or black. After the reaction, the solution was stored in a beaker in an ice bath to keep the solution cool, and then deionized (DI) water and HCl (35%, OCI) were sequentially added to adjust the pH to 7. Next, the solution was filtered with water and ethanol to remove the remaining Li^+^, Cl^−^, and EDA. After that, the filtered powder was dried in a vacuum oven for a few hours. For the control experiments, each single crystal structure of TiO_2_, including only the anatase phase and only the rutile phase, were also prepared.

### Characterization of the samples

To investigate the crystallinity of the rTiO_2_ materials, including their crystal orientations and morphologies, the prepared samples were characterized by X-ray diffraction (XRD; Rigaku Ultima IV), transmission electron microscopy (TEM; JEOL JEM-2100F), and Raman spectroscopy with a laser wavelength of 532 nm. To investigate the energy bandgap properties and diffuse reflectance spectra, the UV-3600 and UV-VIS-NIR spectrophotometers (Shimadzu Corp.) were used. In addition, BaSO_4_ was used as a standard. To investigate the chemical properties, X-ray photoelectron spectroscopy (XPS; ESCA 2000, VG Microtech) and electron spin resonance (ESR; X-band CW-EPR, QM09, RT, 2.97 mW, 9.64 GHz microwave frequency, 100 KHz modulation frequency, modulation amplitude: 1G, powder: 5 G, 10G ESR spectral data) measurements were obtained from the Korea Basic Science Institute in Seoul, Korea.

### Photocatalytic degradation of algae

**Figure 9:**
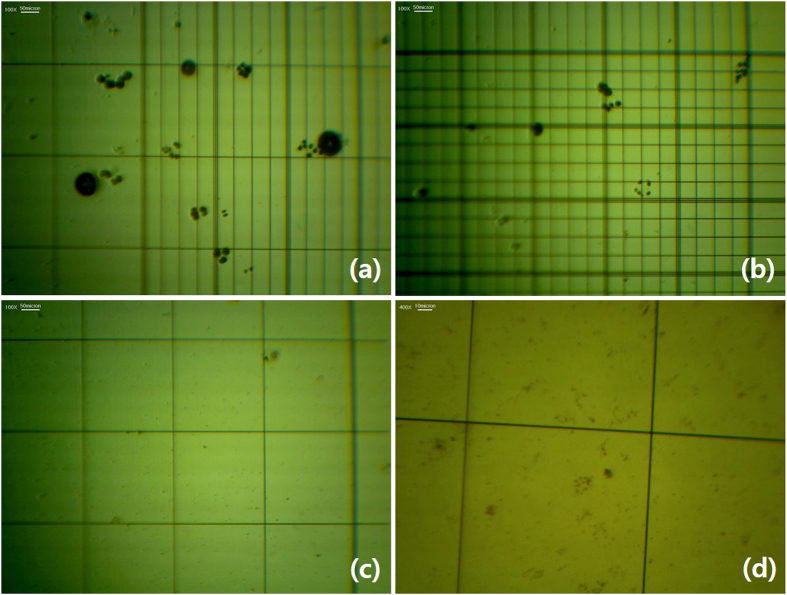
Optical microscope images with haemacytometers of algae that are alive (**a,b**) and that have been fully eliminated (**c,d**).

The investigation of the photocatalytic degradation of algae was carried out in petri dishes that each contained a 5-ml suspension of *Chlamydomonas segnis* (*Lobochlamys segnis,* NLP Co., Ltd.), in which the initial concentration was 7500 ± 10 cells/ml. Next, 10 mg (0.125 mmol) of each TiO_2_ sample in 20 ml of water were added and then stirred at room temperature (25 °C) for four hours while being irradiated by a UV lamp (300–500 nm, POWER ARC UV 100, UV Process Supply, Inc.) or solar simulator (150 Watt Ushio 150-MO Ozone Free Arc Lamp, HS Technologies). For every 30-min interval, 20 μl of the suspension was collected to monitor and analyze the cell conditions. The living algae cells showed random movement, but the dead algae did not move; instead, changes in the cell shapes and sizes were observed with an optical microscope (BiMeince Corp.) and a haemacytometer (Marienfeld-Superior) (Fig. 9). The living algae cells were counted three times for each sample to get the average number of cells. There were no changes in pH during the photocatalytic degradation.

## Additional Information

**How to cite this article**: Kim, Y. *et al.* Solar-light photocatalytic disinfection using crystalline/amorphous low energy bandgap reduced TiO_2_. *Sci. Rep.* **6**, 25212; doi: 10.1038/srep25212 (2016).

## Supplementary Material

Supplementary Information

## Figures and Tables

**Table 1:** Algae elimination reaction conditions^a^.

Entry	Catalyst
1	Only Algae^b^
2	Algae + P-TiO_2_
3	Algae + P6-rTiO_2_
4	Algae + R-TiO_2_
5	Algae + R6-rTiO_2_
6	Algae + A-TiO_2_
7	Algae + A6-rTiO_2_

^a^Reactions were carried out with each sample (10 mg, 0.125 mmol) in 20 ml of water with 4 h of stirring at room temperature under UV or solar irradiation.

^b^5 ml of suspension were added; initial concentration was 7500 ± 10 cells/ml.
