# Supplementary material for: Solar-light photocatalytic disinfection using crystalline/amorphous low energy bandgap reduced TiO2
Source: Sci Rep. 2016 Apr 28;6:25212. doi: 10.1038/srep25212 (PMC4848476; doi:10.1038/srep25212)
Supplement: Supplementary Information [file srep25212-s1.doc]

Supporting Information for

Solar-light photocatalytic disinfection using crystalline/amorphous low energy bandgap reduced TiO_2_

**Youngmin Kim, Hee Min Hwang, Luyang Wang, Ikjoon Kim, Yeoheung Yoon and Hyoyoung Lee***

Center for Integrated Nanostructure Physics, Institute for Basic Science, Department of Chemistry and Department of Energy Science, Sungkyunkwan University, Suwon 440-746, Korea
*Corresponding author. E-mail: [hyoyoung@skku.edu](mailto:hyoyoung@skku.edu)

Fax: +82-031-290-5934; tel: +82-031-299-4566.


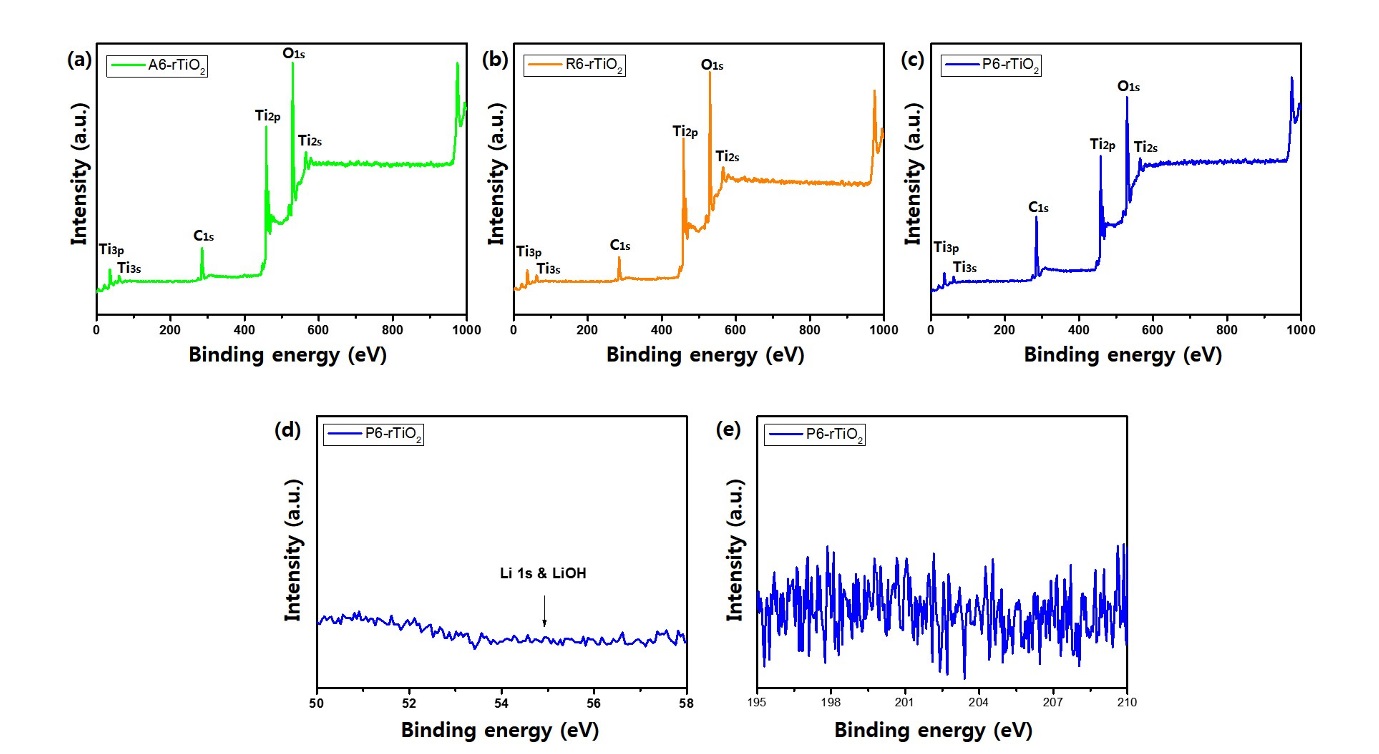


**Figure S1.** XPS survey scan spectra of reduced TiO_2_ samples. (a) anatase phase only TiO_2_ after six days of being reduced (A6-rTiO_2_), (b) rutile phase only TiO_2_ after six days of being reduced (R6-rTiO_2_), (c) P25 TiO_2_ after six days of being reduced (P6-rTiO_2_), and (d, e) are Li 1s and Cl spectra of P6-rTiO_2_, respectively.


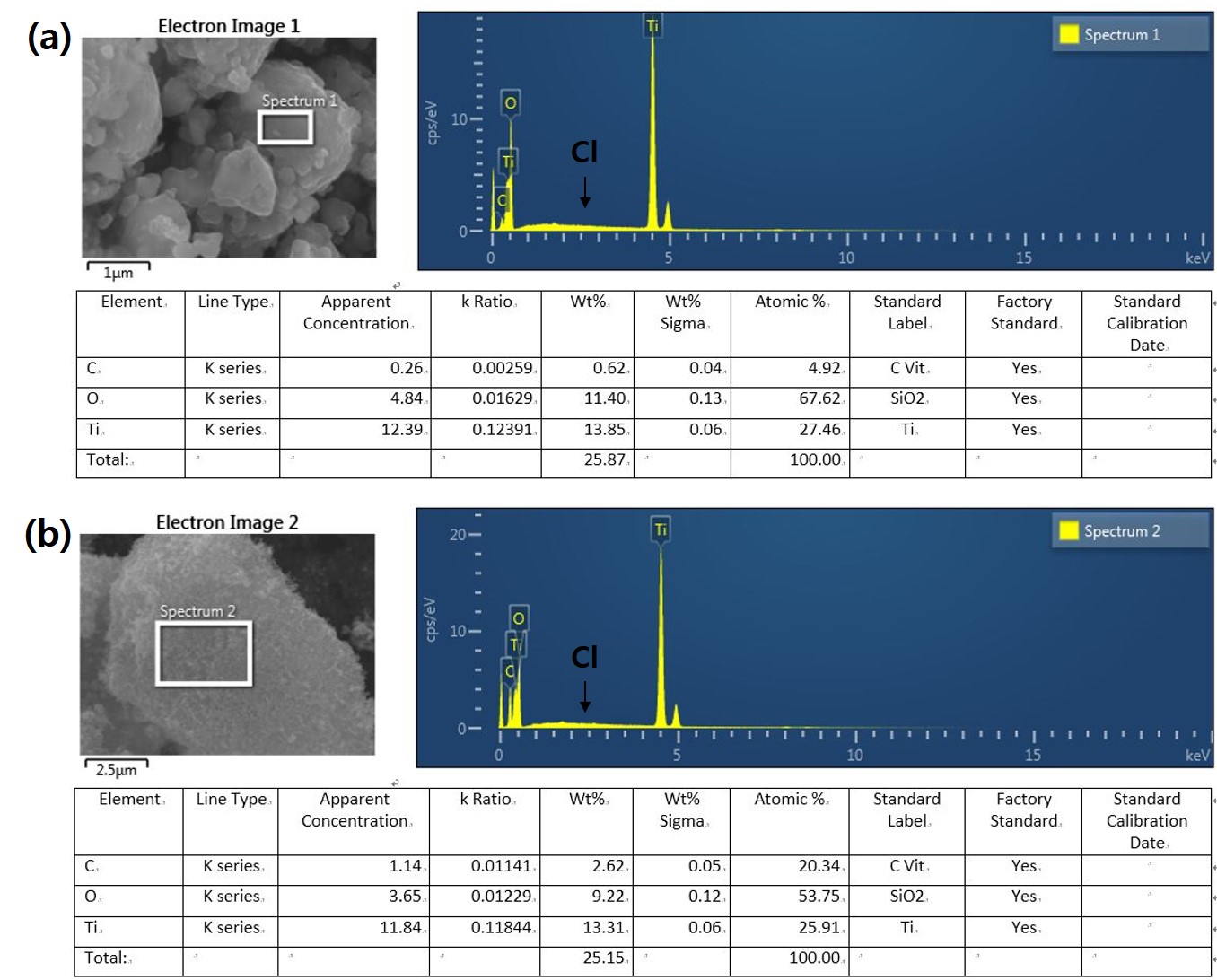


**Figure S2.** EDS analysis of (a) R6-rTiO_2_ and (b) P6-rTiO_2_.


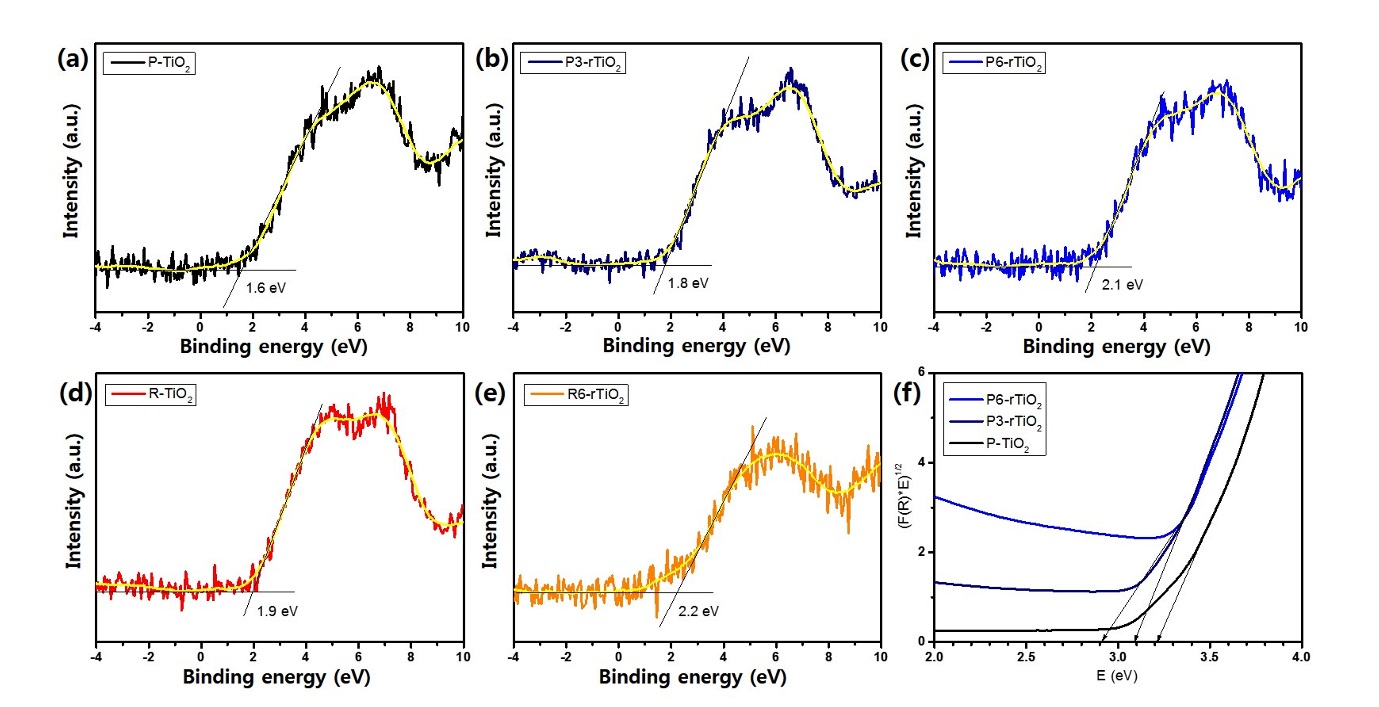


**Figure S3.** Valence band XPS spectra of TiO_2_ samples, (a) pristine P25 TiO_2_ (P-TiO_2_), (b) P25 after 3days of being reduced (P3-rTiO_2_), (c) P25 TiO_2_ after six days of being reduced (P6-rTiO_2_), (d) rutile phase only TiO_2_ (R-TiO_2_) and (e) rutile phase only TiO_2_ after six days of being reduced (R6-rTiO_2_). (f) The diffuse reflectance spectra of P25 TiO_2_ and its reduced samples.


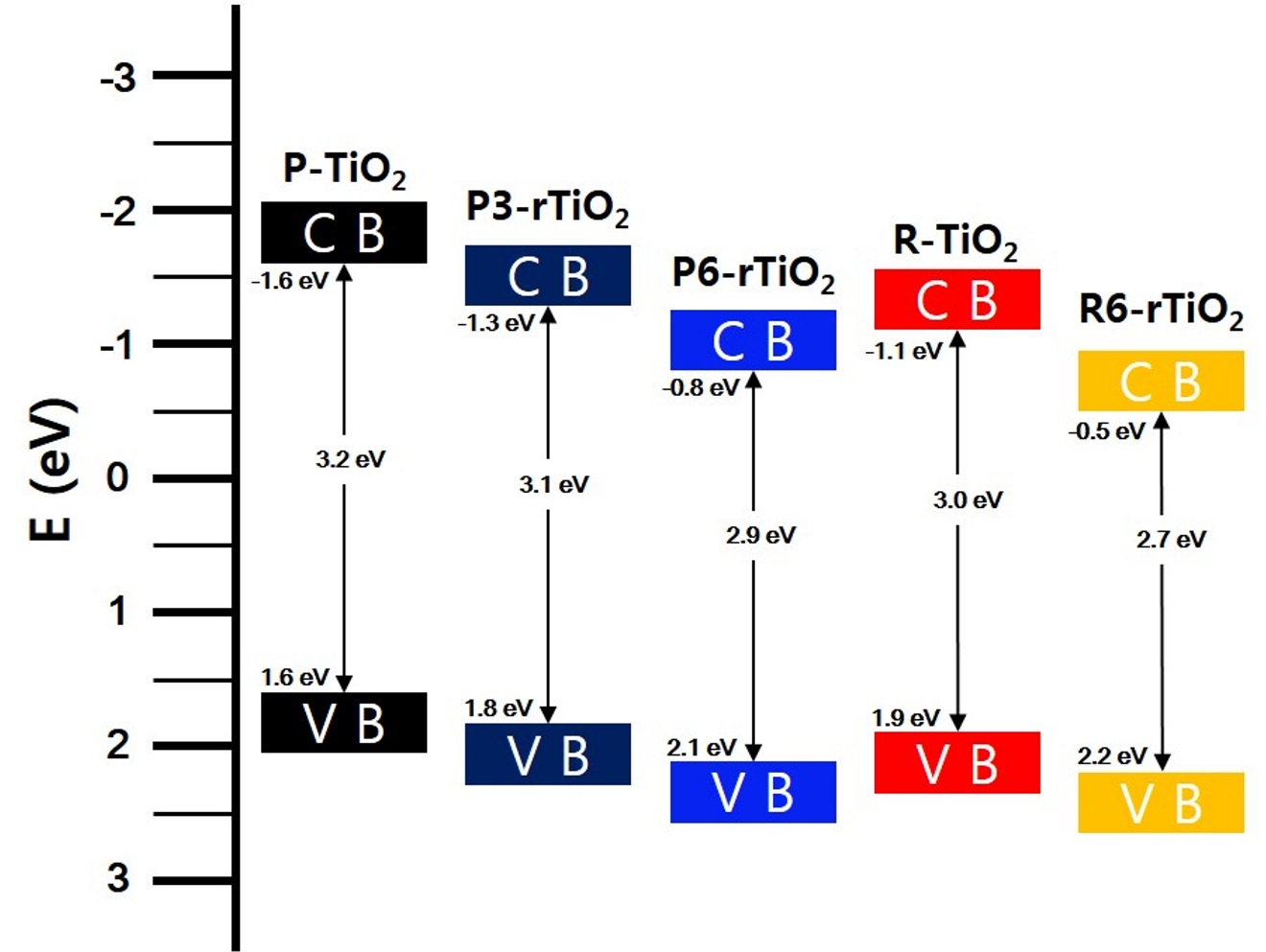


**Figure S4.** The proposed band diagrams of TiO_2_ and reduced TiO_2_ samples.
